# Simultaneous Quantification of Trehalose and Trehalose 6-Phosphate by Hydrophilic Interaction Chromatography/Electrospray Accurate Mass Spectrometry with Application in Non-Targeted Metabolomics

**DOI:** 10.3390/molecules28083443

**Published:** 2023-04-13

**Authors:** Ye Tao, Yannick Rossez, Clovis Bortolus, Luminita Duma, Faustine Dubar, Franck Merlier

**Affiliations:** 1 Sorbonne Universités, Université de Technologie de Compiègne, Génie Enzymatique et Cellulaire (GEC), UMR-CNRS 7025, CS 60319, 60203 Compiègne Cedex, France; 2 CNRS, UMR 8576—UGSF—Unité de Glycobiologie Structurale et Fonctionnelle, Université de Lille, 59655 Lille, France; 3 INSERM U1285, Université de Lille, CHU de Lille, UMR CNRS 8576—UGSF—Unité de Glycobiologie Structurale et Fonctionnelle, 59000 Lille, France; 4 Université de Reims Champagne-Ardenne, CNRS, ICMR UMR 7312, 51097 Reims, France

**Keywords:** trehalose, trehalose 6-phosphate, sugar, sugar phosphate, high resolution time-of-flight MS, HILIC

## Abstract

High-resolution mass spectrometry (HRMS) was coupled with ultra-high-performance liquid chromatography (UHPLC) to simultaneously quantify trehalose and trehalose 6-phosphate without derivatization or sample preparation. The use of full scan mode and exact mass analysis also makes it possible to carry out metabolomic analyses as well as semi-quantification. In addition, the use of different clusters in negative mode makes it possible to compensate for deficiencies in linearity and inerrant saturation at time-of-flight detectors. The method has been approved and validated for different matrices, yeasts, and bacteria, and has shown differentiation between bacteria as a function of growth temperatures.

## 1. Introduction

**Figure 1:**
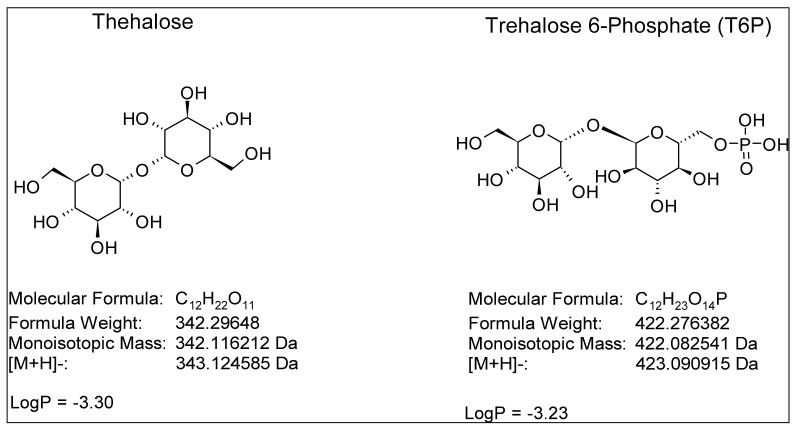
Structure and properties of trehalose and trehalose 6-phosphate.

Trehalose, a disaccharide, and its intermediate, the trehalose 6-phospahate (T6P) (Figure 1), appear to play a crucial role in resistance to stresses, like water [1], salt [2], or temperature [3], for different organisms. To obtaina deeper understanding of the role of these metabolites, it is necessary to be able to quantify it reliably at sufficiently low detection thresholds. The current analysis methods offer either the quantification of sugars or phosphate sugars, and sometimes, sugar and phosphate sugars simultaneously by anionic chromatography [4]. Trehalose, like most sugars, can be quantified by enzymatic methods [5] or by liquid chromatography (LC) with different separation mechanisms such as normal phase separation [4], hydrophilic interaction liquid chromatography (HILIC) [6], or by ion chromatography [7]. As part of the LC coupling, different types of detections were implemented such as refractive index measurement (RI), an evaporative light scattering detector (ELSD) [8], amperometric measurement [4,7], mass spectrometry in tandem (MS/MS), accurate mass measurement (HTMS) [9], and by positive [10,11] or negative electrospray ionisation. After derivatization, it is also possible to quantify it by gas chromatography with flame ionization detection (FID) or by mass spectrometry (MS) with electronic ionization (EI). On the other hand, the charge and the strong polarity of T6P make its analysis complex. T6P can be analyzed by high-performance anion-exchange chromatography (HPAEC) with conductimetric or MS detection [12], HILIC-MS [12,13,14], and a reverse phase after a derivatization step [15]. At the same time, it has been shown that the data acquired by HILIC separation can be used for non-targeted metabolomic analysis using HRMS [16,17,18]. The objective of this work is to develop a versatile method for the simultaneous determination of trehalose and T6P, but also to be able to use the data acquired in the context of non-targeted metabolomic [11,18] and fluxomic analyses [19]. Our previous work has shown that it is possible to use a high-resolution time-of-flight MS (MS-TOF) in conjunction with a HILIC chromatographic separation to perform sugar separations [20]. We demonstrate herein that it is possible to obtain simultaneous quantitative information on sugars and phosphate sugars and, at the same time, to generate data allowing the characterization of samples by metabolomics without derivatization in order to remain compatible with fluxomic analyses [19].

## 2. Results and Discussion

### 2.1. LC-HRMS Method Development and Validation

The choice of the HILIC separation seems relevant due to the strong polarity of trehalose and T6P. The separations are distinguished by the presence of a stationary phase polar group and a mobile phase with a high percentage of buffered acetonitrile [21]. The ionic character of the phosphate group led us to choose a zwitterionic interaction with the column to promote the separation of the phosphate sugars [13,16,17,22]. If the separation made by ion chromatography requires dedicated instrumentation, HILIC chromatography can be performed on a conventional HPLC system. In addition, the use of a mobile phase based on water and acetonitrile makes the coupling with the source of ionization possible by electrospray. In comparison, IC requires a desalting system because of the use of a mobile phase very heavily loaded with salts. The composition of the mobile phase plays a key role in the HILIC separation, whether it is the ionic charge to promote the separation or the choice of salts and possibly a volatile acid to fix the pH [23]. While MS/MS analysers attach to a given cluster, [M + H]^+^, [M − H]^−^, or [M − HCO_2_]^−^, it is possible to simultaneously monitor different clusters by HRMS without loss of sensitivity due to the simultaneity of the detection of a MS-TOF (Table 1).

**Figure 2:**
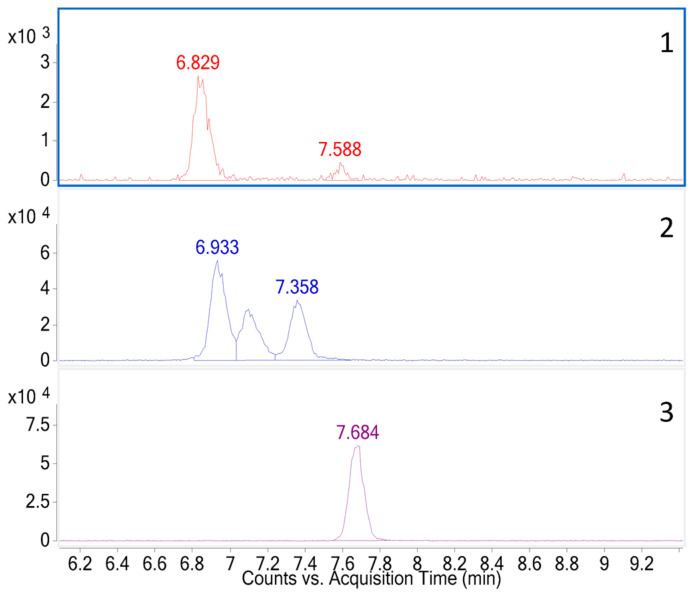
Extracted ion chromatogram of TFA cluster of sucrose (1), sucrose and maltose (2), and ^13^C_12_ trehalose (3) of negative electrospray HILIC chromatography.

**Figure 3:**
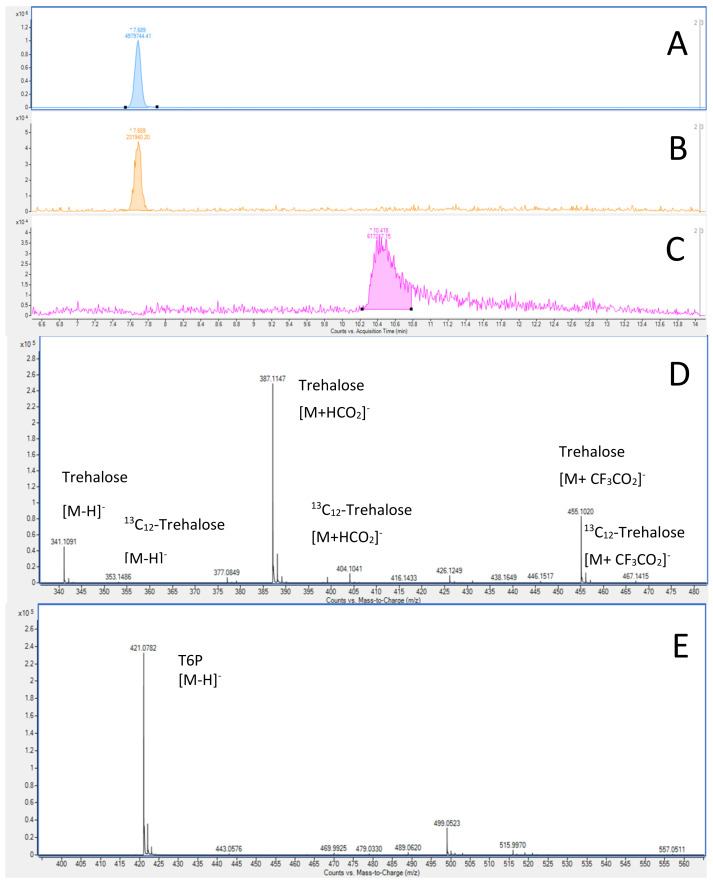
Extracted ion chromatogram of [M − H]^−^ cluster of trehalose (**A**), ^13^C_12_ trehalose (**B**), and T6P (**C**), their ESI-HRMS spectra for trehalose, its internal standard (**D**), and for T6P (**E**). * Saturation of TOF detector.

The pH of the mobile phase plays an essential role both in the separation of carbohydrates and their ionization. In order to simultaneously quantify the two molecules, our initial choice focused on the use of formic acid and ammonium formate at 25 mM to develop our separation. For that, it is necessary to make sure to separate the different isomers and to avoid an overlapping of the peaks (Figure 2). The use of an isotopically ^13^C labelled standard for trehalose improved both the determination of the retention time of trehalose and the precision of the quantitative analysis. The separation of the trehalose isomers as well as the symmetry of the peaks were satisfactory (Figure 3). The T6P peak presents a small peak tailing (Figure 3C) characteristic of a secondary interaction with the metal parts of the column body but also of the HPLC system, probably due to the phosphate group [24]. In 2021, Agilent marketed a new column where this concern was partially corrected but which can only give its full potential with a bio-compatible HPLC system. The same year, Waters also offered HPLC systems and HPLC columns under the "Premier" brand to reduce its secondary interactions. 

Analysis of negative electrospray spectra shows the presence of several clusters, [M − H]^−^ at 341.1089 u, [M + HCO_2_]^−^ 387.1144 u, and more surprisingly [M + CF_3_CO_2_]^−^ at 455.1018 u for trehalose and the equivalent ions for the internal standard (Table 1). At the same time, we observed the cluster [M − H]^−^ independently of the conditions or the concentrations used. Although the mobile phase does not contain TFA, residual traces of this ion-pair agent may remain in the fluidic circuit of the HPLC but also in the electrospray source leading to the formation of this adduct. During the production of the trehalose calibration curves, it was observed that the ratio between the clusters did not have the same dynamics, which reinforces the interest of using an isotopically labelled internal standard for higher accuracy. The fluctuation of the abundance of the different clusters renders the use of the [M − H]^−^ cluster of ^13^C_12_-trehalose as an internal standard for the quantification of T6P irrelevant. In the absence of a perfect internal standard such as T6P labelled with ^13^C or another sugar phosphate, we opted for an external calibration.

**Figure 4:**
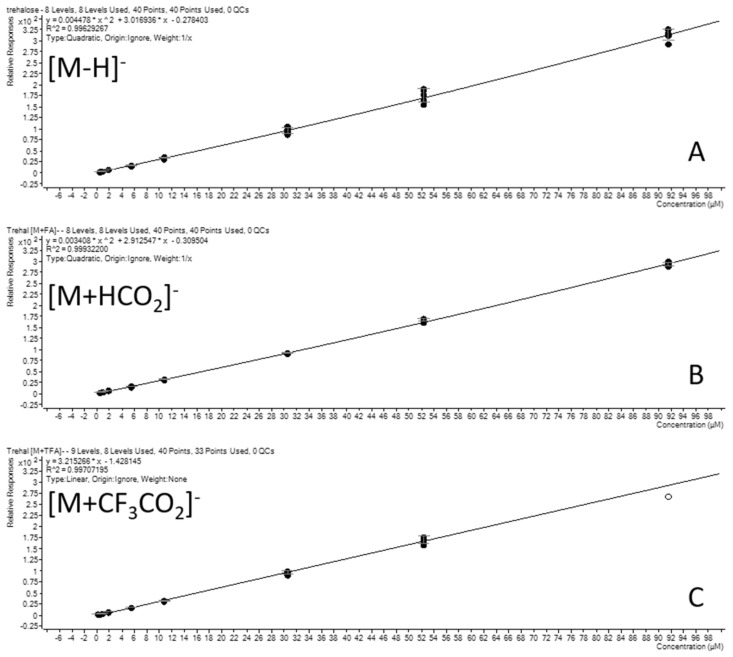
Calibration curve of trehalose monitored by (**A**): [M − H]^−^; (**B**): [M − HCO_2_]^−^; (**C**): [M − CF_3_CO_2_]^−^.

Although the calibration curves of the three clusters are linear (Figure 4), it appears that the area of the cluster [M − CF_3_CO_2_]^−^ has a larger absolute value than in the descending order from the cluster [M + HCO_2_]^−^ to [M − H]^−^. Through the ratio with the internal standard, we observed similar values for the three clusters, except at the end of the calibration curve (Table 2). Due to a better ionization efficiency for the TFA cluster, the latter tends to saturate beyond 30 µM. Conversely, in low concentrations, the signal to noise decreases rapidly from 1 µM for the cluster [M − H]^−^. These characteristics suggest that in the low concentration range (1–10 µM), it is preferable to carry out the quantification of trehalose from the TFA cluster with a decrease in the limit of quantification (LOQ). Conversely, at significant trehalose concentrations (i.e., greater than 50 µM), the use of the cluster [M + H]^−^ should be preferred in order to consider the upper LOQ and limit the saturation effect of the TOF detector.

In this specific case, the use of ^13^C-labelled T6P would be of great help, but the latter is not commercially available and should be chemically synthesized [14]. In this study, we decided to use an external calibration curve for the T6P in order to limit the cost of the analyses. During the work, we followed the synthetic sample value to verify the accuracy of the results. We quickly noticed a drop in the ionization yield after the injections of a series of samples. This problem was solved by adding a step to send the HPLC flow to the waste bin before the retention time of trehalose and after T6P. This compensation is possible within the framework of a targeted quantification but was limited to the first minutes of the separation within the framework of non-targeted metabolomic study.

### 2.2. LC-HRMS Method Validation

#### 2.2.1. Linearity, Limit of Detection (LOD), and Limit of Quantification (LOQ)

A linearity higher than 0.999 is obtained for trehalose with the internal standard on the three main clusters. The LOD/LOQ are, respectively, 1 µM and 0.5 µM with [M − H]^−^ clusters. LOQ is 0.5 µM for [M − CF_3_CO_2_]^−^ (Figure 4). Regarding the T6P, external and internal calibration curves using ^13^C_12_-trehalose were produced. In real conditions, too much trehalose tends to drastically reduce the ionization yield in a differentiated way from T6P. We therefore preferred to use an external calibration carried out periodically during long series of analyses. In this case, the linearity was 0.995 for 1 to 50 µM with significant peak tailing. The LOD/LOQ was 1 µM/5 µM (Figure S2). 

#### 2.2.2. Robustness, Method Precision, and Trueness

During the development of the chromatographic method, we observed significant fouling from the electrospray source. To remedy this, we used the 6-way valve upstream of the QTOF to minimize to penetration towards the mass spectrometer. This led to the incoming HPLC eluent stream being sent to the wastebasket before the elution time of 6 min and after 14 min. If the approach gives good results in the context of a quantitative analysis, it may present the risk of omitting some analytes for non-targeted analytes intended for metabolomics. We carried out several injections of the points of the calibration curves and compared the results of different quality control (QC) points (Table 3). The trueness of point was below 10% for each point and the precision below 3%. No noticeable change in accuracy is observed at initial day (D0) and after 6 days (D6) for trehalose (Table 4) and after 4 days (D4) for T6P (Table 5). The monitoring of the precision and accuracy of the QC was carried out at different stages of the development of the chromatographic method, in particular before the increase in the flow rate and the optimization of the gradient ramps, then during the passage of 92 measurement points. The robustness of the method could thus be demonstrated both on the retention times and on the accuracy of the method over a period of 48 h.

In the case of trehalose, the results remain within the measurement deviation of less than 1.7 ± 0.6% thanks to the use of an internal standard (Figure S2). Conversely, a modification of the calibration curve is observed due to the fouling of the ionization source not compensated by an internal standard (Figure S3) with a variation of 10.5 ± 2%. It was also possible to notice a clear improvement in the areas at one day (D1) compared to D0 thanks to the source cleaning and calibration step and tune of the mass spectrometer.

### 2.3. LC-HRMS Quantification and Metabolomic Analysis of Acinetobacter baumannii Samples

**Figure 5:**
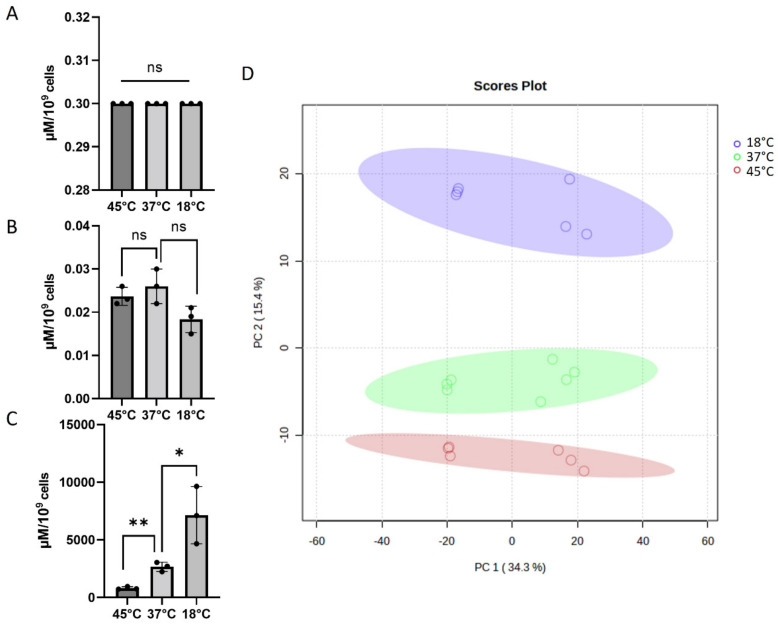
Trehalose (**A**), T6P (**B**), S6P (**C**) quantification of *Acinetobacter baumannii* AB5075 grown at 45, 37, and 18 °C, respectively. PCA scores plot shows variances in metabolites (994 unassigned molecules) extracted from *A. baumannii* grown at the three indicated temperatures (**D**). The results correspond to n = 3 biologically independent samples for A, B, and C. The analysis was performed using MetaboAnalyst V5.0 (https://www.metaboanalyst.ca/, accessed on 2 March 2023). Statistical significances were determined by a two-tailed student’s *t* test **, *p* ≤ 0.005; *, *p* ≤ 0.05; ns; non-significant.

Trehalose, T6P, and sucrose-6P (S6P) were quantified on lysed *A. baumannii* grown at three different temperatures—45 °C, 37 °C, and 18 °C—to test the method in complex samples. Surprisingly, no variations were observed for trehalose (Figure 5A) while some variations were found for the T6P but without significance (Figure 5B). These results are explained by a time of growth leading to decreased bacterial cells viability as described for *Escherichia coli* [25]. In the same study, the bacteria were grown a few hours during the cold treatment and the authors have shown a clear decrease in intracellular trehalose content proportionally with cell viabilities. In our conditions, the bacteria were grown overnight (i.e., more than 10 h) and therefore for a longer period than in the latter paper. Interestingly, S6P was more informative, with concentrations varying greatly between the conditions and globally higher than for trehalose and T6P (Figure 5C). The highest concentration was found at 18 °C with a significant decrease at 37 °C and with the same trend at 45 °C. However, sucrose was barely detected leading to the hypothesis of the S6P role as thermal protectants of *A. baumannii* or as a source of energy to cope with the low temperature. More work will be needed to answer this question, but other carbohydrates, including sucrose, have been shown to play a role as non-accumulated osmoprotectants in *Sinorhizobium meliloti* [26]. The role for trehalose as an osmo- and thermoprotectant is well accepted, and it could be the case for S6P as well. 

To complete this targeted quantification, 994 unassigned metabolites were compared by principal component analysis (PCA) at the three temperatures investigated herein (Figure 5D). Each sample is shown as a single point with a 95% confidence interval ellipse representing the range of the data. All temperatures have large confidence ellipses. This statistical data investigation clearly demonstrates the power of our method to work on targeted and non-targeted metabolism analyses.

## 3. Experimentals

### 3.1. Chemicals

Solvents, formic acid, and ammonium formate were purchased from Biosolve Chimie with UPLC-MS grade (Dieuze, France). Maltose, sucrose, trehalose, and T6P were purchased from Sigma-Aldrich (St. Quentin Fallavier, France). α,α [UL-13C12] trehalose (^13^C_12_-trehalose) was purchased from Santa Cruz Biotechnologie (Dallas, TX., USA). Buffers were prepared with Milli-Q water, purified using a Milli-Q system (Millipore, Molsheim, France). 

### 3.2. Biological Material

#### 3.2.1. Yeasts

Two or three colonies of *Candida albicans* SC5314 were stirred overnight in 5 mL of RPMI medium. The next day, the pre-culture was added in 100 mL of RMPI medium and a culture kinetic study was initiated at an OD = 0.2 until the exponential stage was reached at OD = 0.8 (about 3 h). The culture was divided in two aliquots in order to apply the different growing conditions. The culture was left for 1 h at 37 °C with stirring. After this time, one sample (control) was left at 37 °C. In another sample (oxidative stress), 0.5 mM of peroxide of hydrogen was added. After 1 h at 37 °C and with stirring, the number of cells of the samples were counted on a Thomas cell. Based on these results, part of the sample was taken and diluted 3 times to obtain a 100 µL solution with 100 cells. This solution was spread on Sabouraud-type agar and left to incubate overnight. The rest of the sample was placed at 4 °C. The next day, a count of the living cells on the agars was carried out, which makes it possible to obtain a percentage of viable cells. This percentage was then applied to the total sample and allowed to determine the volume necessary to obtain 8 × 10^6^ cells from each sample. The removed culture was centrifuged at 500 *g* for 20 min, the supernatant was eliminated, and the pellet was heated at 100 °C for 1 h before being stored at −25 °C. 

#### 3.2.2. *Acinetobacter baumannii*

The strain AB5075 was incubated in 30 mL at 18 °C, 37 °C, and 45 °C overnight at 180 rpm in lysogeny broth (LB) Miller. The metabolites extraction was performed as follow based on Hayner et al. [27] After centrifugation of the 30 mL of the overnight cell suspensions (3000 *g* for 10 min), the cell pellets were washed with PBS twice. After resuspending the cells, they were lysed in pure water at 100 °C for 20 min. After centrifugation at 4 °C for 10 min at 16,000 *g*, the supernatants were ready for analyses or kept at −20 °C if needed later. The bacterial density was measured before the lysis to report the metabolite quantities per cell numbers.

### 3.3. Internal Standard Preparation

^13^C_12_-trehalose was accurately weighted by high-precision balance and then dissolved in distilled water to obtain a 100 mg/mL IS-1 stock solution stored at 4 °C.

### 3.4. Standard and QC Samples Preparation

Trehalose and T6P were accurately weighted by high-precision balance and then dissolved in distilled water to obtain a 10 mg/mL stock solution stored at 4 °C. The standard solution (0.5, 1, 2, 5, 10, 30, 50, 100 µM) was prepared by diluting the stock solution with distilled water continuously. The standard calibration samples were performed by adding 10 μL of IS-1 solution into 90 μL of each calibration solution. The QC points were obtained by preparing two points at 2 and 20 µM (low and medium, respectively) concentration from two other independent weightings. The standards and QC samples were handled according to the same sample processing steps as unknown samples.

### 3.5. Sample Preparation

After thawing, 10 μL of the IS-1 solution was added to 90 μL of each sample before being injected into the LC-MS systems.

### 3.6. LC-HRMS Instrumentation and Analytical Conditions

Trehalose and T6P quantification was performed by LC-HRMS on an HPLC Agilent 1290 with DAD connected to Agilent Q-TOF 6538. HPLC was carried out on an Agilent Poroshell 120 HILIC-Z (100 × 2.1 mm ID, 2.7 µm) column connected to an Agilent Infinity 1290 HPLC. The solvent system was A: 25 mM of ammonium formate and 0.1% formic acid in H_2_O and B: Acetonitrile. The gradient program began and stayed with 97% B during 1 min, then ramped to 60% B at 13 min, increased to 30% B in 2 min, held at 30% during 2 min, returned to the initial conditions, and kept constant for 3 min. The flow rate was 0.500 mL/min and injection volume was 4 µL. All compounds response was measured in ESI- and calibrated externally. The ESI Gas Temp was 300 °C, Vcap − 3000 V; the Drying Gas was set at 12 L/min and Nebuliser at 30 psig. The Fragmentor was set at 135 V. The HRMS spectrum was register at 2 Hz in the mass range of 100 to 1200 *m*/*z* with internal calibration. The LC flux was sent to the mass spectrometer between 6 and 14 min and to the trash the end of the time.

### 3.7. Validation

#### 3.7.1. Selectivity

Selectivity was ensured by monitoring the retention time of trehalose and of the isotopically labelled internal standard, but also by ascertaining the isomers of trehalose (sucrose, maltose). Due to the absence of commercially available labelled T6P and the lack of other diphosphates, only monitoring the exact mass of the different isotopomers of T6P [13,23] as well as the stability of the retention time in two different matrices allowed us to conclude on the selectivity.

#### 3.7.2. Linearity and LLOQ

Individual standard stock solutions were diluted and evaluated. LOQ values were determined for each compound at an S/N of 10. An additional evaluation of simultaneous IS quantification trehalose and T6P by ^13^C-trehalose was performed.

#### 3.7.3. Accuracy and Precision

The accuracy was evaluated by monitoring QC sample during several days after the passage of 92 samples. Due to the complexity of the matrix, it was not possible to reproduce it without the presence of trehalose or T6P.

#### 3.7.4. Stability

The stability of the method was evaluated by following the quantification and retention time of QC during a series of 100 yeast samples and during a sequence of 18 samples (3 injections each) from the extraction of *Acinetobacter baumannii*.

### 3.8. Data and Statistical Analysis

MassHunterB07 software suit (Agilent) and MSdial [28] were used for data processing, which was performed as described previously with some modifications [29]. Briefly, Agilent generated files (*.d) were converted into the *.mzML format using MSConvert and followed by MS-DIAL version 4.8 [28]. MetaboAnalyst 5.0 [30] was used to estimate variation across the sample group (PCA). For each sample, the peak area of each metabolite was normalized to the total peak areas. Graphs were made using Prism Software V 9.0. The results were considered significant for a *p* value of ≤0.05. Between three and six assays were carried out for each temperature.

## 4. Conclusions

This study demonstrates the feasibility of the trehalose and T6P measurement up to 1 µM.

The use of a time-of-flight analyser, generating high-resolution mass spectra in full acquisition mode, also allows the data thus accumulated to be used posteriori for non-targeted processing. Beyond the simple target quantification of the two metabolites, this method also enables using the data for metabolomic analyses, making it possible to highlight particular conditions and opening the way to semi-quantification of other molecules as we have done here for S6P if additional questions arise after the statistical analyses. The versatility of this separation could be the first choice for non-targeted metabolomic analyses. The chromatographic method can be further improved by the joint use of the most recent column technologies and HPLC equipment to reduce the peak tailing of sugar phosphates.

## Figures and Tables

**Table 1:** Exact mass and retention time of the different clusters of trehalose and T6P.

Target	Formula	[M − H]^−^	[M + HCO_2_]^−^	[M + CF_3_CO_2_]^−^	RT (min)
Trehalose	C_12_H_22_O_11_	341.1089	387.1144	455.1018	7.6
^13^C_12_-trehalose	^13^C_12_H_22_O_11_	353.1492	399.1547	467.1421	7.6
T6P	C_12_H_23_O_14_P	421.0753	467.0807	535.0681	10.3

**Table 2:** Reponses of trehalose at different concentrations (µM) for clusters [M − H]^−^; [M − HCO_2_]^−^, and [M − CF_3_CO_2_]^−^. Signal to noise (S/N), RIS: Internal standard relative response.

[Trehalose] (µM)	[M − H]^−^			[M + HCO_2_]^−^			[M + CF_3_CO_2_]^−^		
	Area	S/N	RIS	Area	S/N	RIS	Area	S/N	RIS
0.5	8313	3	1.35	55,156	54	1.14	328,667	72	1.13
1	23,466	Infinity	2.37	148,429	90	2.23	683,028	142	2.31
2	45,477	24	5.39	303,672	77	5.52	1,124,959	166	5.56
30	656,765	123	86.33	4,166,579	563	89.82	4,647,415	268	89.92
90	1,607,254	229	292.1	10,082,234	768	299.14	4,716,700	23	267.23

**Table 3:** Intra-day assay precision and trueness data for trehalose (3 levels) for 2 LC conditions and T6P (1 level) at final LC condition. Trueness % = (Observed concentration − nominal concentration)/(nominal concentration) × 100.

Id		Conc. Target (µM)	Average Conc. (µM)	Standard Deviation	RSD	LC Slow	LC Fast	Trueness
QC2	Trehalose	22.45	20.88	0.54	2.60%	8	5	6.98%
QC3		7.84	7.12	0.11	1.50%	0	5	9.18%
QC5		2.57	2.36	0.03	1.29%	9	0	8.23%
QC2	T6P	7.84	7.29	0.25	3.36%	0	5	8.23%

**Table 4:** Intra-day assay and extra-week (day 0, 1, and 6) precision of calibration curve of trehalose: RIS = (Area of Trehalose)/(Area of ^13^C_12_-trehalose).

	Ris					Intra-Day			Extra-Week		
Conc. (µM)	D0	D1	D6 r1	D6 r2	D6 r3	Average	Standard Deviation	RSD	Average	Standard Deviation	RSD
0.46	1.14	1.10	1.09	1.08	1.06	1.08	0.02	1.42%	1.09	0.03	2.74%
0.91	2.23	2.22	2.21	2.20	2.27	2.23	0.04	1.58%	2.23	0.03	1.14%
1.81	5.52	5.27	5.21	5.31	5.46	5.33	0.12	2.32%	5.36	0.13	2.40%
5.55	14.37	14.48	14.95	14.60	14.56	14.70	0.21	1.44%	14.59	0.22	1.49%
10.77	31.03	30.53	31.70	31.39	31.79	31.63	0.21	0.67%	31.29	0.52	1.65%
30.53	89.82	90.93	91.82	91.89	89.48	91.06	1.37	1.50%	90.79	1.11	1.22%
52.33	161.21	167.33	165.28	165.02	169.15	166.48	2.31	1.39%	165.60	2.97	1.79%
91.58	299.14	291.69	288.28	294.58	291.64	291.50	3.15	1.08%	293.07	4.06	1.39%

**Table 5:** Intra-day assay and intra-week (day 0, 2, and 4) precision of calibration curve of T6P.

Conc. (µM)	Area (External Cal.)				Intra-Day			Intra-Week		
	D0	D2	D4 n1	D4 n2	Average	Standard Deviation	RSD	Average	Standard Deviation	RSD
0.55	9080	10,476	10,590	9377	9984	858	8.59%	9881	765	7.74%
1.09	19,783	16,930	20,161	23,334	21,747	2244	10.32%	20,052	2621	13.07%
2.71	48,540	54,242	58,297	63,093	60,695	3391	5.59%	56,043	6173	11.02%
5.28	100,434	109,706	123,374	121,699	122,537	1185	0.97%	113,803	10,793	9.48%
10.01	204,196	195,637	230,506	255,039	242,773	17,347	7.15%	221,345	26,921	12.16%
27.75	530,722	540,559	636,781	640,689	638,735	2764	0.43%	587,188	59,678	10.16%
55.50	1,103,943	1,113,634	1,310,572	1,358,745	1,334,659	34,063	2.55%	122,1723	131,940	10.80%
111.00	3,187,728	3,669,272	3,897,902	3,983,834	3,940,868	60,763	1.54%	3,684,684	356,911	9.69%

## Data Availability

Not applicable.

## Supplementary Materials

The following supporting information can be downloaded at: https://www.mdpi.com/article/10.3390/molecules28083443/s1, Figure S1: External calibration curve of T6P forum 0.5 to 25 μm; Figure S2: Variation of the Trehalose calibration curve over time between 0 and 6 days; Figure S3: Variation of the external calibration curve of T6P during a week.

## Sample Availability

Samples of the compounds for yeast and *Acinetobacter baumannii* are available from the authors.
