# Supplementary material for: Simultaneous Quantification of Trehalose and Trehalose 6-Phosphate by Hydrophilic Interaction Chromatography/Electrospray Accurate Mass Spectrometry with Application in Non-Targeted Metabolomics
Source: Molecules. 2023 Apr 13;28(8):3443. doi: 10.3390/molecules28083443 (PMC10145281; doi:10.3390/molecules28083443)
Supplement: Supplementary file 1 [file molecules-28-03443-s001.zip › molecules-2314693-supplementary.pdf]

Simultaneous quantification of trehalose and trehalose 6-phosphate by hydrophilic interaction chromatography/electrospray accurate mass spectrometry with application in non-targeted metabolomics – supplementary information.

Author names and affiliations.

Ye Tao [a], Yannick Rossez[a,b], Clovis Bortolus [b], LuminitaDuma[a,c],FaustineDubar[b]& Franck Merlier[a]

Addresses

[a] Sorbonne Universités, Université de Technologie de Compiègne, Génie Enzymatique et Cellulaire (GEC), UMR-CNRS 7025, CS 60319, 60203 Compiègne Cedex, France

[b]INSERM U1285, Univ. Lille, CHU de Lille, UMR CNRS 8576 - UGSF - Unité de Glycobiologie Structurale et Fonctionnelle, Lille, 59000, France
[c] Université de Reims Champagne-Ardenne, CNRS, ICMR UMR 7312, 51097 Reims, France

Corresponding author.

Franck MERLIER, Email address: franck.merlier@utc.fr, Tel +33 (0) 3 44 23 73 55

Figure SI-1:External calibration curve of T6P forum 0.5 to 25µm.
Figure SI-2: Variation of the Trehalose calibration curve over time between 0 and 6 days.
Figure SI-3:Variation of the external calibration curve of T6P during a week.

Figure SI-1

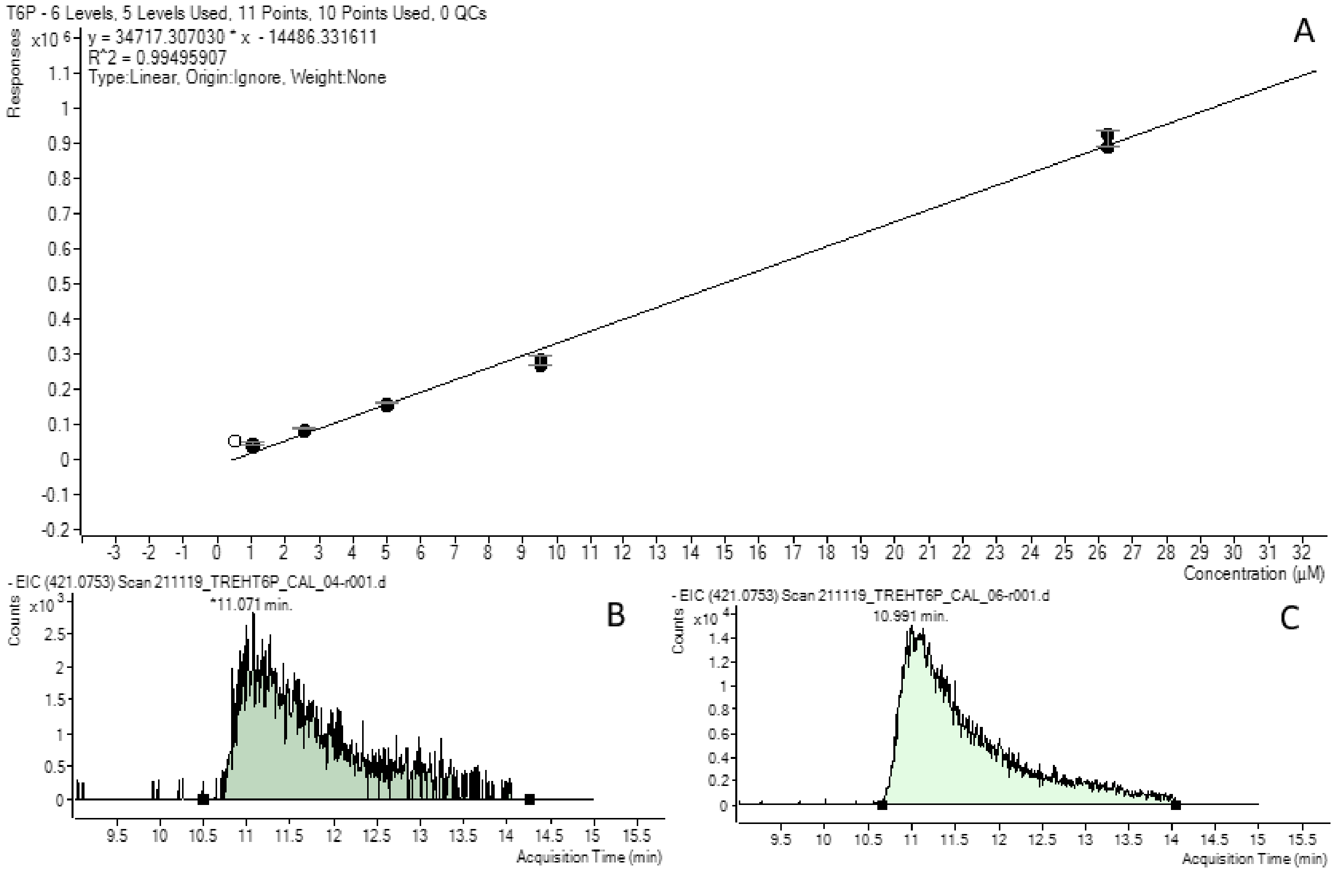

External calibration curve of T6P forum 0.5 to 25 µM (A) based on extracted ion chromatogram(XIC) of ions [M-H]-, 421.0753 ± 100 ppm and response at 5 (B) et 26.2 µM (C)

Figure SI-2: Variation of the Trehaloseinternal calibration curve over time between 0 (Blue), 1 (red) and 6 days (green).

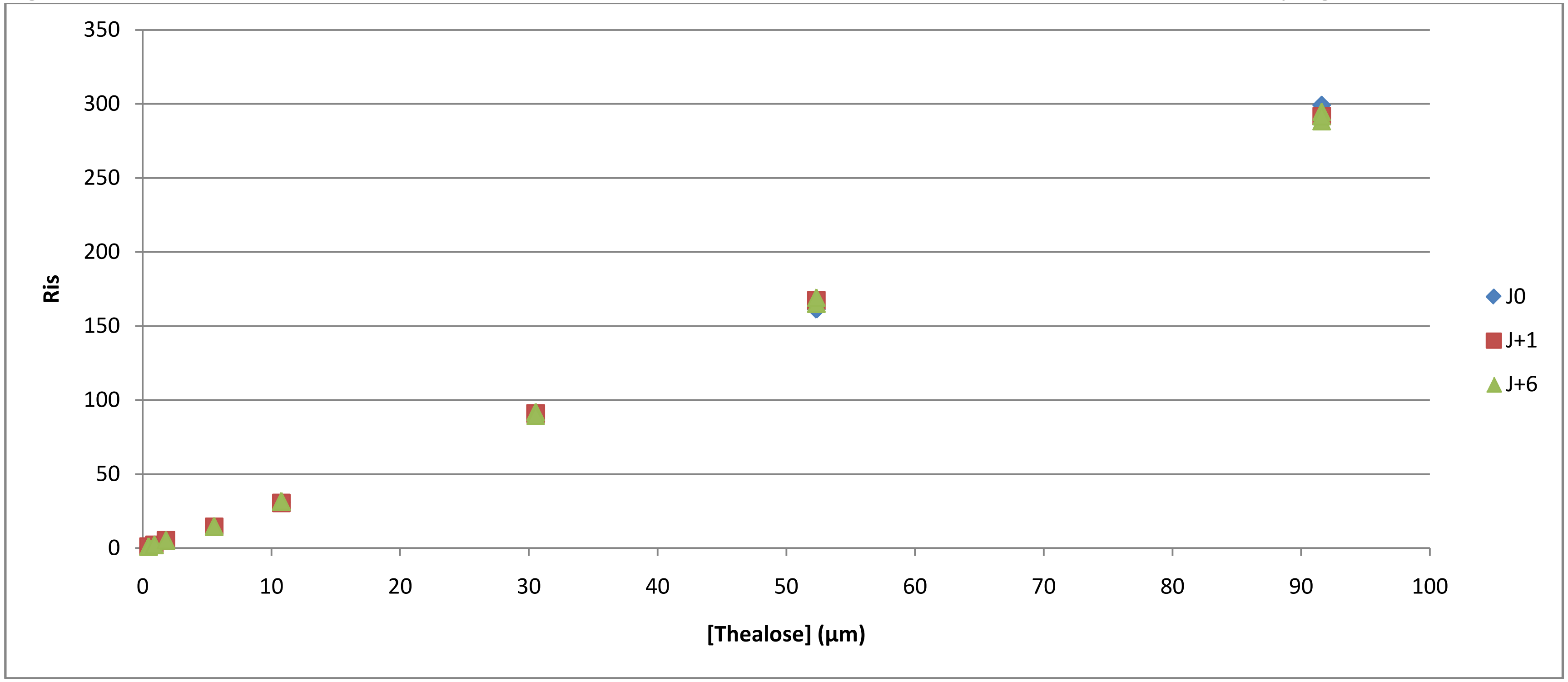

RIS = (Area of Trehalose)/(Area of $^{13}C_{12}$-Trehalose). J0/J1: 1 replicate, J6: 2 replicates.

Figure SI-3: Variation of the external calibration curve of T6P during a week.

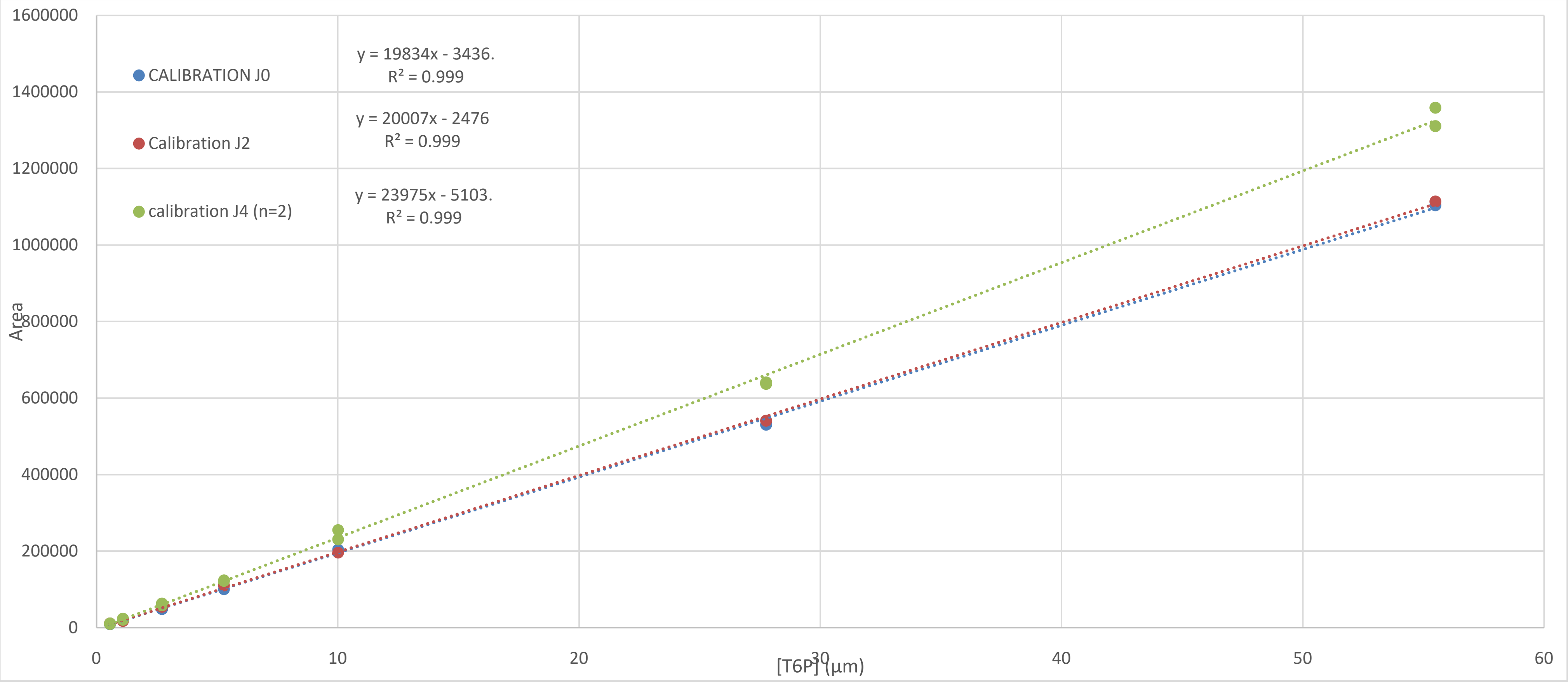

The injection of the first range point and the last are separated by 13 hourswithout calibration and it is separated by 38 injections including also QC.Calibration 1 (blue) at T=0, n=1; Calibation 2 (red) at T=33h, n=1; Calibation 3 (green) at T=80h, n=1
